# Optimizing Water–Fertilizer Coupling Across Different Growth Stages of Tomato in Yellow Sand Substrate: Toward Enhanced Yield, Quality, and Resource Use Efficiency

**DOI:** 10.3390/plants14060936

**Published:** 2025-03-17

**Authors:** Yalong Song, Jiahui Xu, Shuo Zhang, Jianfei Xing, Long Wang, Xufeng Wang, Can Hu, Wentao Li, Zhanming Tan, Yunxia Cheng

**Affiliations:** 1 College of Mechanical and Electrical Engineering, Tarim University, Alar 843300, China; 10757232242@stumail.taru.edu.cn (Y.S.); 10757231181@stumail.taru.edu.cn (J.X.); 10757241148@stumail.taru.edu.cn (S.Z.); 120140002@taru.edu.cn (L.W.); wxf@taru.edu.cn (X.W.); 120140004@taru.edu.cn (C.H.); 120230009@taru.edu.cn (W.L.); 2 Xinjiang Production and Construction Corps Key Laboratory of Utilization and Equipment of Special, Alar 836500, China; 3 Modern Agricultural Engineering Key Laboratory, Universities of Education Department of Xinjiang Uygur Autonomous Region, Alar 843300, China; 4 College of Engineering, China Agriculture University, Beijing 100083, China; 5 College of Horticulture and Forestry Sciences, Tarim University, Alar 843300, China; tlmdxtzm@taru.edu.cn (Z.T.); chengyunxia2018@163.com (Y.C.)

**Keywords:** yellow sand substrate cultivation, deficit irrigation, fertilization at different growth stages, water–fertilizer coupling, comprehensive evaluation

## Abstract

The tomato (*Solanum lycopersicum* L.) is widely cultivated in yellow sand substrate-based systems in Northwest China, contributing significantly to regional agriculture. However, suboptimal water and fertilizer management hinders the balanced optimization of yield, fruit quality, and resource efficiency. In this two-year solar greenhouse experiment (2023–2024), we employed a four-factor, three-level orthogonal design [L9(3^4^)] to examine three irrigation regimes—full irrigation (FI 100% of crop evapotranspiration, [ET_c_]), mild deficit irrigation (DIM 75% ET_c_), and severe deficit irrigation (DIS 50% ET_c_)—in combination with staged fertilizer applications at the seedling, flowering/fruit-set, and peak-fruit stages. A multi-objective decision-making framework, integrating an improved entropy weight method with a virtual-ideal-solution-based TOPSIS model, was established to address the complexity of water–fertilizer interactions. The results indicated that irrigation amount (IA) was the primary determinant of yield and water use efficiency (WUE), followed by fertilizer application in the second stage (FII). For fruit quality indices (moisture content, vitamin C (VC), lycopene (LC), soluble sugars (SSs), and soluble solids content (SSC)), IA remained the most influential factor, followed by FIII, FII, and FI; IA also had the largest impact on fruit hardness (Hd), soluble protein (SP), and titratable acidity (TA). An integrated scoring analysis revealed that treatment T5 achieved the highest yield, WUE, and partial factor productivity (PFP), whereas T7 excelled in fruit quality (VC, LC, SSC, and SP). Moreover, TOPSIS confirmed T5 as the optimal water–fertilizer strategy to achieve high yield, improved quality, and efficient resource utilization. Overall, these findings underscore a robust approach for optimizing water–fertilizer coupling in tomato cultivation under yellow sand substrate conditions, thereby enhancing resource use efficiency, promoting sustainable greenhouse agriculture in arid regions, and contributing to national water-saving and yield-increasing priorities.

## 1. Introduction

Tomato (*Solanum lycopersicum* L.) is a globally significant fruit and vegetable crop, playing a critical role in sustainable agricultural systems and global food security [1]. According to the Food and Agriculture Organization (FAO, 2022), global tomato cultivation covers 4.92 million hectares, with an annual production of 186 million tons. China, as the world’s leading producer and consumer of tomatoes, accounts for 1.14 million hectares of cultivated land and 68 million tons of annual production [2]. Despite its economic importance, suboptimal water and fertilizer management in yellow sand substrate cultivation systems remains a major constraint, significantly limiting tomato yield, fruit quality, and resource use efficiency.

Irrigation and fertilization are critical factors influencing tomato growth and development. Scientific irrigation practices and stage-specific water–fertilizer management can enhance nutrient absorption and utilization efficiency, thereby improving crop yield, fruit quality, and resource use efficiency. A well-designed irrigation strategy regulates nutrient absorption and transfer, significantly influencing photosynthesis and transpiration [3]. The unique physical properties of yellow sand substrates allow for rapid water and nutrient infiltration, preventing root zone saturation and optimizing gas exchange [4]. Moderate deficit irrigation (e.g., 75% ET_c_) stabilizes yield while enhancing fruit quality, improving fruit set, productivity, and reducing physiological disorders [5,6]. However, excessive deficit irrigation (e.g., 50% ET_c_) reduces the net photosynthetic rate and negatively affects yield [7]. Studies show that moderate irrigation can balance water conservation and yield improvement goals by promoting starch breakdown, increasing fructose content, and accumulating dry matter in fruits, albeit with slight reductions in VC and carotenoids [8,9]. Furthermore, moderate irrigation decreases fruit Hd and TA, enhancing taste and market competitiveness [10].

Fertilization timing and method significantly influence tomato physiological activity, growth characteristics, and nutrient conversion [11]. Compared to traditional methods, drip fertilization with a 10–30% reduction in fertilizer inputs shows minimal differences in yield [12]. Stage-specific nitrogen application has been shown to meet the plant’s precise nutrient demands at various growth stages, improving nitrogen use efficiency and yield [13]. For example, targeted nitrogen application during vegetative growth promotes photosynthesis and biomass accumulation, while potassium application during flowering and fruit-setting enhances sugar accumulation and stress resistance [14]. Studies on various crops, including tomatoes [15], maize [16], and rice [17], confirm that stage-specific fertilization improves yield, fertilizer use efficiency, and fruit quality by meeting nutrient demands more effectively. Water–fertilizer coupling has been widely recognized for its synergistic impact on tomato growth. Increased irrigation and nitrogen application significantly enhance leaf nitrogen content, while precision fertilization systems, which synchronize water and nutrient delivery to the root zone, further improve fertilizer use efficiency [18,19]. Proper fertilization management optimizes nutrient concentrations and enhances fruit quality by maintaining nutrient availability within optimal ranges [20,21]. Despite these advancements, the effects of specific fertilizer ratios at different growth stages on tomato yield, quality, and overall production efficiency in yellow sand substrate systems remain poorly understood. Therefore, optimizing water–fertilizer coupling to improve yield and quality during various growth stages requires further in-depth research.

This study hypothesizes that integrating optimal fertilization rates with appropriate irrigation volumes at specific growth stages can synergistically enhance tomato yield, fruit quality, and water–fertilizer use efficiency in yellow sand substrate cultivation systems. The objectives of this study are: (1) To comprehensively evaluate the effects of varying irrigation volumes and fertilization rates across different growth stages on tomato yield, fruit quality, and water–fertilizer use efficiency under yellow sand substrate cultivation. (2) To determine the relative importance of evaluation factors using an improved entropy weight method and assess the overall impact of water–fertilizer management through the integration of the improved TOPSIS method with a virtual ideal solution. (3) To identify the optimal irrigation-fertilization strategy tailored to yellow sand substrate systems, providing robust scientific evidence for high-quality, high-yield tomato production, and efficient resource utilization in desert and arid regions.

## 2. Materials and Methods

### 2.1. Site Description and Materials

**Figure 1:**
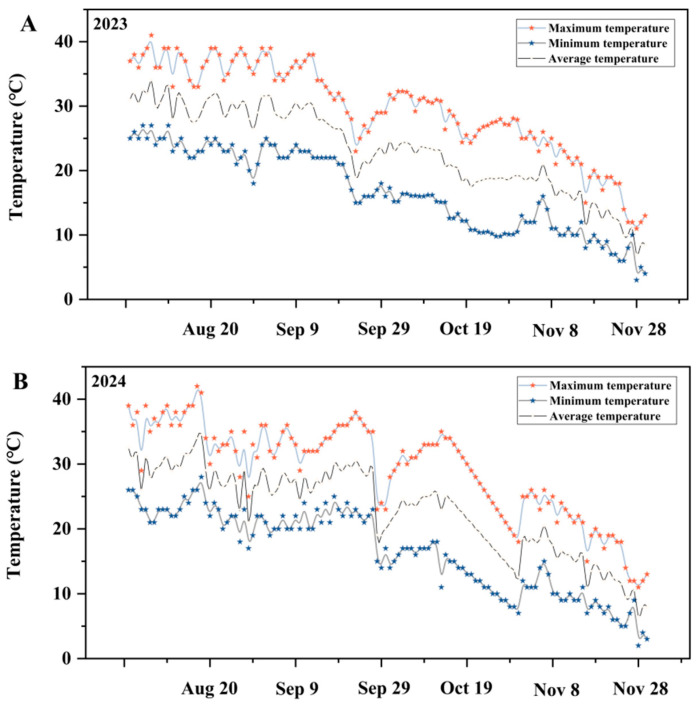
(**A**,**B**) Temperature variations in the greenhouse during the cultivation periods of 2023 and 2024.

A two-year solar greenhouse experiment (2023–2024) was conducted in a solar greenhouse located at Aksu Naida Agricultural Technology Co., Ltd., Aksu, Xinjiang, China (40.97680° N, 80.12610° E; altitude 1106 m, soil pH 8.2). By controlling environmental factors such as temperature and humidity inside the greenhouse, the stability and controllability of experimental conditions are ensured. The greenhouse, measuring 120 m in length, 15 m in width, and 6 m in ridge height, was equipped with a real-time temperature monitoring system integrated with a smart thermometer (China San Sheng Smart Agricultural Technology Co., Ltd., Zhangjiakou, China). Detailed temperature data collected throughout the cultivation process are presented in Figure 1. The physical and chemical properties of the yellow sand substrate used across the two growing seasons are summarized in Table 1.

The tomato variety used for the experiment was “Ling guan 705”, a cultivar renowned for its adaptability and strong market demand in local facility agriculture. Seedlings, each with three leaves and one heart, were transplanted at a spacing of 0.4 m between plants and 0.6 m between rows. Single-stem pruning was applied, with each plant retaining five clusters of fruit. Water and fertilizer management commenced 15 days after transplanting. The experiment was conducted in late summer based on the climatic characteristics of Xinjiang and the growth cycle of tomatoes. After transplanting tomatoes in late summer, they can better adapt to the local temperature and light conditions, while avoiding the more severe climatic variations in spring, thus, enhancing the reliability and reproducibility of the experiment. In 2023, transplanting was conducted on 3 August. The seedling stage lasted from 3 August to 31 August, followed by the flowering and fruit-setting stage from 1 September to 15 October, and the fruit-bearing stage from 16 October to 30 November. In 2024, transplanting was performed on 9 August, with the seedling stage lasting from 9 August to 5 September, the flowering and fruit-setting stage from 6 September to 10 October, and the fruit-bearing stage from 11 October to 7 December.

### 2.2. Experimental Design

The study employed a four-factor, three-level orthogonal experimental design [L9(3)^4^]. This experiment was conducted using a completely randomized block design to investigate four factors: irrigation amount (IA), fertilizer application rate at the seedling stage (FI), fertilizer application rate at the flowering/fruit-setting stage (FII), and fertilizer application rate at the peak-fruit stage (FIII). Three irrigation levels were tested: severe deficit (DIS, 50% ET_c_), mild deficit (DIM, 75% ET_c_), and full irrigation (FI, 100% ET_c_). Based on recommended rates for water-soluble compound fertilizers, three fertilizer levels (562.5, 750, and 937.5 kg ha^−1^) were selected. In total, nine treatments were established, each replicated three times to ensure a uniform distribution of factor levels across the experiment.

#### 2.2.1. Plot Specifications and Isolation Measures

This experiment employed a completely randomized block design with three replicates per treatment, resulting in 27 plots. Each plot measured 3 m in row spacing by 10 m in row length, covering an area of 30 m^2^, and was planted with 50 tomato plants per row at a spacing of 0.2 m. To prevent lateral water and fertilizer movement, a 0.1 mm-thick plastic film was inserted vertically to a depth of 40 cm between adjacent plots. Preliminary dye-tracing tests [22] confirmed that this physical barrier effectively blocked cross-plot seepage. Within the greenhouse, uniform lighting and ventilation were maintained, and treatments were randomly distributed to minimize local environmental variations.

#### 2.2.2. Evapotranspiration (ET_c_) Calculation and Irrigation Amount Determination

In this study, irrigation was determined based on crop evapotranspiration (ET_c_) following Gao et al. [23], using the equation *I* = *E_p_* × *K_cp_* × *A*, where *I* is the irrigation amount, *E_p_* is the cumulative evaporation (mm) from a standard evaporation pan, *K_cp_* is the pan coefficient, and *A* is the irrigated area (m^2^). A standard evaporation pan (20 cm in diameter, 10 cm in depth) was placed at canopy height in the greenhouse, and water-level changes were recorded regularly. To accurately determine *K_cp_*, we calibrated pan evaporation data against the actual substrate moisture dynamics and crop water consumption under sandy substrate conditions at the seedling, flowering/fruit-setting, and peak-fruit stages. Following this field calibration, the final *K_cp_* values for these three stages were set at 0.6, 1.3, and 1.1, respectively. The irrigation water source was local groundwater, and the electrical conductivity of the irrigation water was 0.5 dS/m.

### 2.3. Water–Fertilizer Integration System and Fertilizer Ratio

#### 2.3.1. Management of Intelligent Drip Irrigation and Fertilization System

The experiment utilized an intelligent drip irrigation and fertigation system within a greenhouse for precise irrigation and fertilization management. The system comprises drip tapes placed along the root zones of plant rows, coupled with an automatic control system that provides water and nutrients based on the predetermined daily irrigation amount and fertilizer concentration. Each dripper has a flow rate of approximately 2 L/h, with dripper spacing and layout designed to ensure uniform water distribution across the entire root zone. Irrigation occurs once or twice daily. To ensure accurate irrigation for each treatment, flow meters are installed on the lateral pipes of each treatment and integrated with the control system for real-time monitoring, ensuring that water is applied according to the calculated water requirements.

#### 2.3.2. Fertilizer Ratio

The fertilizer application rates used in this study (562.5, 750, 937.5 kg ha^−1^) refer to the amount applied during each growth season for each growth stage. A fixed fertilizer ratio of N:P_2_O_5_:K_2_O = 20%:20%:20% was adopted for two main reasons. First, balanced fertilization recommended by Xinjiang’s greenhouse tomato cultivation standards [24] meets crop nutrient demands throughout all growth stages and suits the rapid water infiltration characteristic of sandy substrates, thus, preventing salt accumulation due to overuse of a single nutrient [25]. Second, maintaining a fixed nutrient ratio isolates the primary effects of water–fertilizer coupling by minimizing confounding factors introduced by dynamic nutrient adjustments, facilitating a clearer analysis of irrigation and stage-based fertilization interactions. Although tomatoes exhibit varying nutrient requirements at different stages, adjusting the overall application rate rather than the ratio still allowed targeted responses to each developmental phase.

### 2.4. Measurement Results

#### 2.4.1. Physical and Chemical Properties of Yellow Sand Substrate

In this study, ammonium nitrogen (NH_4_^+^-N), available phosphorus (Olsen-P), and available potassium (Available K) were measured by Suzhou Keming Biotechnology Co., Ltd. (Suzhou, China). The maximum field capacity, porosity, and water-holding porosity of the yellow sand substrate were determined using standard soil physics methods (Table 2). Soil bulk density was calculated as the ratio of the dry soil mass to its volume, and pH was measured using a pH meter [26].

#### 2.4.2. Tomato Fruit Yield

Tomato yield was measured for each subplot. Fruits were harvested in multiple batches upon reaching maturity, which was determined based on characteristics such as bright red color, firm and plump shape, smooth surface, and typical aroma. The harvested fruits were then weighed [27], and the yield per unit area was calculated.

#### 2.4.3. Tomato Fruit Quality

To ensure the accuracy of quality indicators, fruit quality was assessed immediately after harvesting at the red-ripe stage. Fruits showing signs of pests or diseases were first removed. The selected fruits were then rinsed with tap water followed by distilled water, and their surfaces were dried with paper towels. Hd was measured using a texture analyzer, and the fruit shape index (FSI) was calculated as the ratio of fruit length to width. VC content was determined using the oxalic acid-EDTA colorimetric method, while SP content was measured via the Coomassie Brilliant Blue colorimetric method. LC was analyzed by spectrophotometry, and TA was quantified through titration. SSs were determined using the anthrone–sulfuric acid colorimetric method, and nitrate (NA) content was measured with an ion-selective electrode. SSC was read with a refractometer, and moisture content (MC) was calculated using the drying-weight method [28].

#### 2.4.4. Water–Fertilizer Use Efficiency

WUE and PFP were calculated using Equations (1) and (2) [29].

(1) $$WUE=\frac{Y}{IA}$$

(2) $$PFP=\frac{Y}{F}$$

In the equations, *WUE* represents water use efficiency (kg m^−3^), *Y* is the fruit yield (kg ha^−1^), *IA* is the amount of irrigation water (m^3^ ha^−1^); *PFP* represents fertilizer partial productivity (kg kg^−1^), and *F* is the fertilization rate (kg ha^−1^). *PFP* refers to the tomato yield obtained per kilogram of fertilizer input.

### 2.5. Data Analysis and Statistics

Data organization and basic calculations were performed using Microsoft Excel (2023). Analysis of variance (ANOVA) and range analysis for the orthogonal experiment results were conducted using SPSS (v23.0). Irrigation and fertilizer amounts were treated as fixed effects, while year and repetitions were included as random effects or covariates to correct for the bias introduced by the imbalance in total fertilizer application. Graphs were created with MATLAB (2023) and Origin (2023). The weights of evaluation factors were calculated based on the results of the multi-criteria orthogonal experiment using the improved entropy method. Subsequently, the impact of water–fertilizer management on the comprehensive production efficiency of tomatoes was systematically evaluated using the improved TOPSIS method with a virtual ideal solution. This methodology provides robust scientific evidence and technical references for optimizing water–fertilizer coupling strategies in tomato cultivation under arid region conditions.

#### 2.5.1. Comprehensive Scoring Method Based on the Improved Entropy Method for Determining Evaluation Factor Weights

The entropy method is an objective weighting approach based on the degree of dispersion of indicator information [30]. The specific steps are as follows: First, the tomato evaluation indicators were standardized. Suppose there are m indicators *X*_1_, *X*_2_, …, *X_m_*, where *X_i_* = {*x_i_*_1_, *x_i_*_2_, …, *x_im_*}. The original data were standardized according to Equation (3) to obtain the standardized indicators *Y_1_*, *Y_2_*, …, *Y_m_*. To address the uncertainty caused by possible zero values in the original data that could affect the entropy calculation, the standardized data were non-negatively shifted by adding 0.01 to each data point, denoted as *Z_ij_*. This preprocessing step effectively avoids the information uncertainty caused by zero values, making the entropy method more robust and reliable when assessing the contribution of each indicator to the overall evaluation result.

(3) $$y_{ij}=\frac{x_{ij}}{\sum_{i=1}^{n}x_{ij}}$$

Next, the entropy value *E_i_* for each indicator was calculated.

(4) $$E_{i}=-k\cdot \sum_{j=1}^{n}y_{ij}lny_{ij}(i=1,2,\cdots ,m)$$

In the equation, *k* = 1/*lnn*; if *y_ij_* = 0, then *y_ij_lny_ij_* = 0. Finally, the weight *H_i_* for each indicator was calculated.

(5) $$H_{i}=\frac{1-E_{i}}{\sum_{i=1}^{n}\left(1-E_{i}\right)}(i=1,2,\cdots ,m)$$

In the weight calculation process, the entropy value (*E_i_*) plays a critical role in determining the weights of each indicator. To enhance the calculation, this study modifies the exponential function in Equation (5) by introducing a variable coefficient (*k*), which adjusts the slope of the tangent to the exponential function. This adjustment allows for controlling the degree of variation in the weights of each indicator. Specifically, with the inclusion of the variable coefficient (*k*), the formula for calculating the weights is expressed as follows:

(6) $$H_{x}=\frac{exp\left[kç\left(\sum_{i=1}^{m}E_{i}-E_{x}+1\right)\right]-exp(kçE_{x})}{\sum_{l=1}^{m}\left\{kç(\sum_{i=1}^{m}E_{i}-E_{l}+1)\right\}-exp(kçE_{l})}$$

In the equation, *x* = 1, 2, …, *m* the value of *k* needs to be adjusted according to the specific problem.

Finally, the comprehensive score is obtained by multiplying the non-negatively shifted data by the weights and summing them, as shown in the following formula:

(7) $$S_{i}=\sum_{i=1}^{m}H_{x}Z_{ij}$$

#### 2.5.2. Improved TOPSIS Method Based on Virtual Ideal Solution

The TOPSIS method evaluates the relative performance of alternative solutions by calculating the distances between each candidate solution and the positive and negative ideal solutions [31]. However, traditional TOPSIS often encounters difficulties in distinguishing the degree of superiority or inferiority among candidate solutions. To address this limitation, virtual positive and negative ideal solutions are introduced. By computing the weighted comprehensive distances between candidate solutions and the positive, negative, and virtual ideal solutions, a closeness coefficient is derived. This approach effectively enhances the discrimination capability of evaluation results. The detailed process is as follows:

1. Construct the normalized matrix. Suppose there are *n* alternatives, each with *m* evaluation indicators. Each indicator is normalized and standardized to obtain the normalized matrix:

(8) $$Z=\left[\begin{array}{cccc} z_{11} & z_{12} & \cdots & z_{1n}\\ z_{21} & z_{22} & \cdots & z_{2n}\\ \vdots & \vdots & \ddots & \vdots \\ z_{m1} & z_{m2} & \cdots & z_{mn} \end{array}\right]$$

2. Determine the positive ideal solution *Z*^+^ and the negative ideal solution *Z*^−^, where the positive and negative ideal solutions are composed of the maximum and minimum values, respectively, from each column of *Z*:

(9) $$\left\{\begin{array}{c} Z^{+}=[max\{z_{11},z_{21},\cdots ,z_{n1}\},\cdots ,max\{z_{1m},z_{2m},\cdots ,z_{nm}\}]\\ =[Z_{1}^{+},Z_{2}^{+},\cdots ,Z_{M}^{+}]\\ Z^{-}=[min\{z_{11},z_{21},\cdots ,z_{n1}\},\cdots ,min\{z_{1m},z_{2m},\cdots ,z_{nm}\}]\\ =[Z_{1}^{-},Z_{2}^{-},\cdots ,Z_{M}^{-}] \end{array}\right.$$

3. Calculate the distance between each alternative and the positive and negative ideal solutions:

(10) $$\left\{\begin{array}{c} D_{i}^{+}=\sqrt{\sum_{j=1}^{m}w_{j}(Z_{J}^{+}-Z_{ij}{)}^{2}}\\ D_{i}^{-}=\sqrt{\sum_{j=1}^{m}w_{j}(Z_{J}^{-}-Z_{ij}{)}^{2}} \end{array}\right.(i=1,2,\cdots ,n)$$

In the equation, *w_j_* represents the comprehensive weight of the *m*-th indicator.

4. Determine the virtual positive ideal solution *V*^+^ and the virtual negative ideal solution *V*^−^, as well as the distances between each alternative and the virtual positive and negative ideal solutions:

(11) $$\left\{\begin{array}{c} V^{+}=2Z^{+}-Z^{-}\\ V^{-}=2Z^{-}-Z^{+} \end{array}\right.$$

(12) $$\left\{\begin{array}{c} F_{i}^{+}=\sqrt{\sum_{j=1}^{m}w_{j}(V_{j}^{+}-Z_{ij}{)}^{2}}\\ F_{i}^{-}=\sqrt{\sum_{j=1}^{m}w_{j}(V_{J}^{-}-Z_{ij}{)}^{2}} \end{array}(i=1,2,\cdots ,m)\right.$$

5. Determine the weighted comprehensive distances between each alternative and the ideal and virtual ideal solutions:

(13) $$\left\{\begin{array}{c} S_{i}^{+}=\alpha D_{i}^{+}+\beta F_{i}^{+}\\ S_{i}^{-}=\alpha D_{i}^{-}+\beta F_{i}^{-} \end{array}\right.$$

In the equation, *α* and *β* are constants, set to 0.9 and 0.1, respectively.

6. Calculate the closeness coefficient *C_i_* of each alternative to the ideal solution.

(14) $$C_{i}=\frac{S_{i}^{-}}{S_{i}^{+}+S_{i}^{-}}$$

In the equation, 0 ≤ *C_i_* ≤ 1, where a value of *C_i_* closer to 1 indicates better overall performance of the alternative.

## 3. Results

### 3.1. Effects of Water–Fertilizer Coupling on Tomato Yield and Water–Fertilizer Use Efficiency

Under the yellow sand substrate cultivation model, the interaction between irrigation and fertilization amounts at different growth stages had statistically significant effects on tomato fruit yield, PFP, and WUE. Experimental results revealed that the T7 treatment group exhibited superior performance, with fruit yield and PFP increasing by an average of 123.01% and 175.46% compared to the lowest yield (T1) and PFP (T3) groups, respectively. Additionally, the T5 group achieved the highest WUE, improving by 105.54% relative to the T1 group. Under both DIS and DIM conditions, varying fertilization amounts at different growth stages significantly affected the two-year average fruit yield, WUE, and PFP, highlighting the sensitivity of these indicators to water availability and nutrient management. However, no significant differences in fruit yield and WUE were observed between the T8 and T9 groups. Overall, at the same fertilization level, FI significantly enhanced fruit yield and PFP by ensuring sufficient water supply, while DIM demonstrated a clear advantage in improving WUE by optimizing water utilization under limited irrigation conditions.

**Figure 2:**
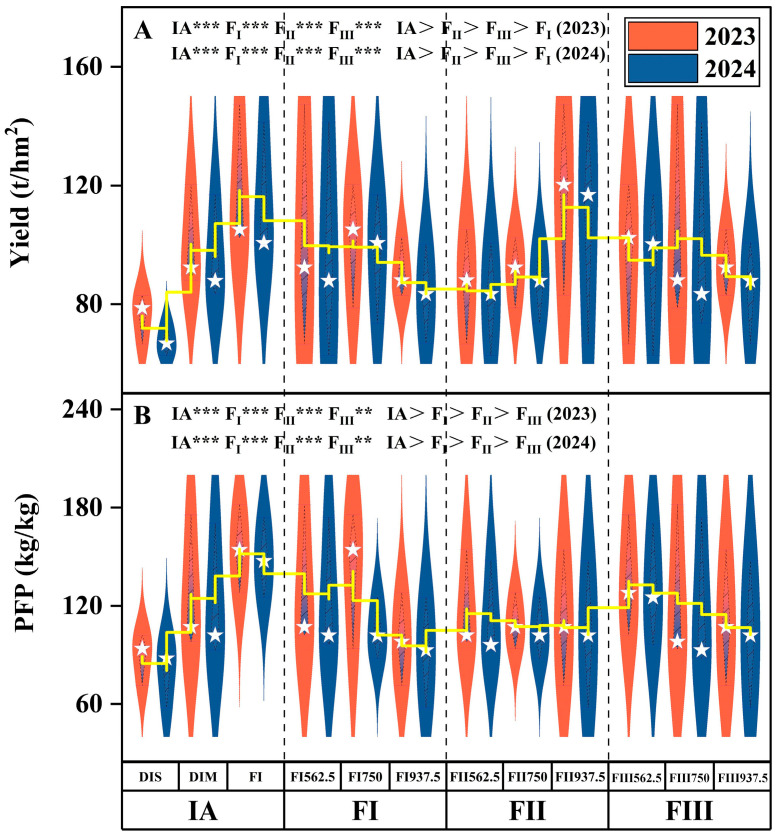
Effects of Different Factor Levels on Tomato Yield (**A**) and PFP (**B**). Note: IA: Irrigation amount; FI: Fertilizer application at seedling stage; FII: Fertilizer application at flowering/fruit-setting stage; FIII: Fertilizer application at peak-fruit stage; DIS: Severe deficit irrigation; DIM: Mild deficit irrigation; FI: Full irrigation.

**Figure 3:**
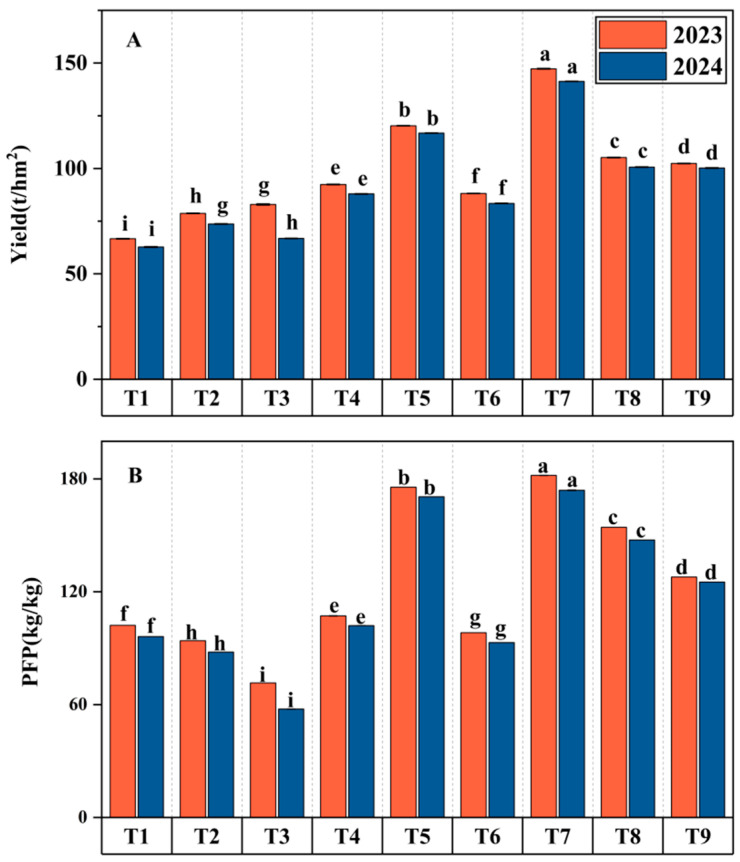
Effects of Different Treatments on Tomato Yield (**A**) and PFP (**B**). Different letters (a–i) indicate statistically significant differences between groups. Groups with the same letter are not significantly different, while groups with different letters show significant differences.

**Figure 4:**
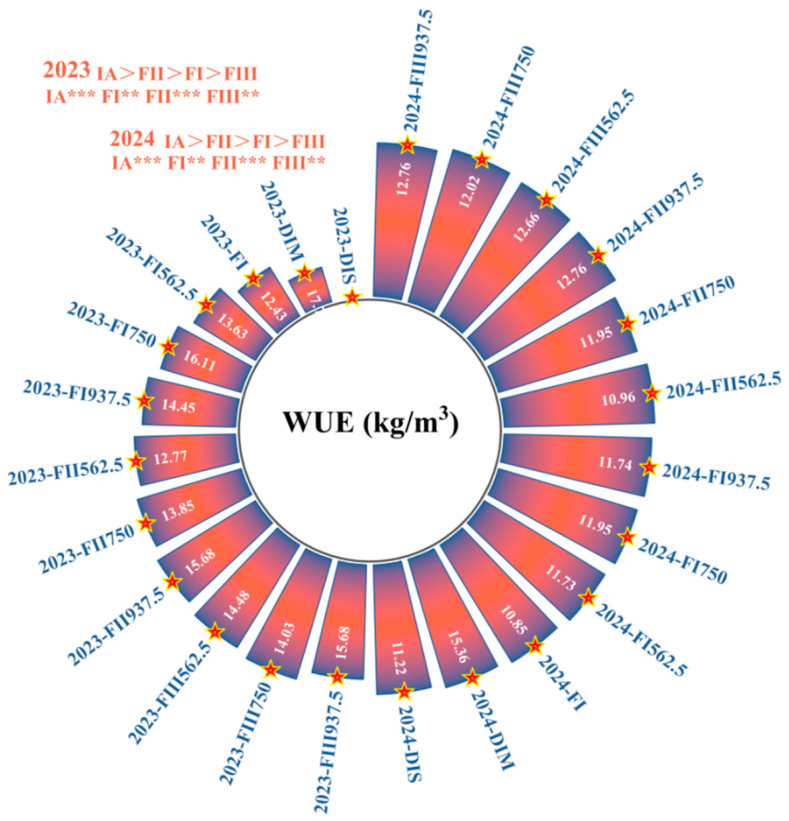
Effects of Different Factor Levels on WUE.

**Figure 5:**
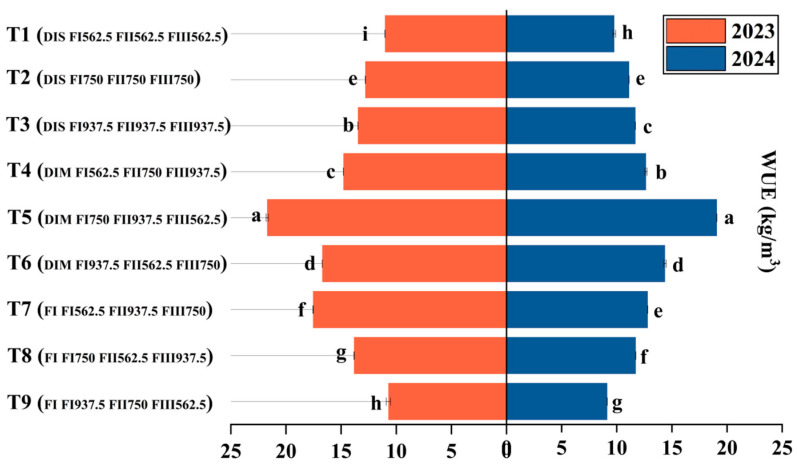
Effects of Different Treatments on WUE. Different letters (a–i) indicate statistically significant differences between groups.

As shown in Figure 2 and Figure 3, range analysis indicated that IA, FI, FII, and FIII all had statistically significant effects on yield, WUE, and PFP (Figure 4 and Figure 5). Among these factors, IA had the most pronounced regulatory effect on all indicators throughout the two-year trials. This was followed by FII and FIII, with FI having a comparatively minor impact. The optimal water–fertilizer combinations for different indicators were identified as follows: Fruit Yield: FI FI 562.5 FII 937.5 FIII 750, WUE: DIM FI 750 FII 937.5 FIII 750, PFP: FI FI 750 FII 937.5 FIII 562.5.

In summary, IA during each growth stage had a more pronounced effect on fruit yield, WUE, and PFP than fertilization alone. When IA differences were not considered, FII had the most significant impact on increasing fruit yield and WUE, while FI showed greater potential for enhancing PFP. The impact of IA amount on yield was particularly notable: FI achieved the highest yield, surpassing DIM by 9.37% and DIS by 26.75%. Compared to FI, DIM reduced average yield and PFP by 15.23% and 17.88%, respectively, but increased WUE by 42.63%. DIS decreased yield and PFP by 35.67% and 42.21%, respectively, while only enhancing WUE by 12.79%. Therefore, DIM, although slightly reducing average fruit yield and PFP, significantly improved WUE, resulting in a more balanced water–fertilizer utilization. In contrast, DIS was less effective in balancing fruit yield and WUE, demonstrating relatively inferior overall performance.

### 3.2. Effects of Water–Fertilizer Coupling on Tomato Fruit Quality

Under the yellow sand substrate cultivation model, the interaction between irrigation amount and fertilization ratio significantly influenced various tomato fruit quality indicators. Based on the two-year average analysis, the T7 exhibited notable improvements in VC, LC, and SSC, with increases of 84.23%, 123.69%, and 36.68%, respectively, compared to the T1. Furthermore, the T4 achieved the highest SS content, which was 122.97% greater than that of the T1, while the T5 group recorded the highest SP content, with an increase of 222.23% over the T1. In contrast, the T1 showed the highest TA levels, whereas the T3 exhibited the highest NA content.

**Figure 6:**
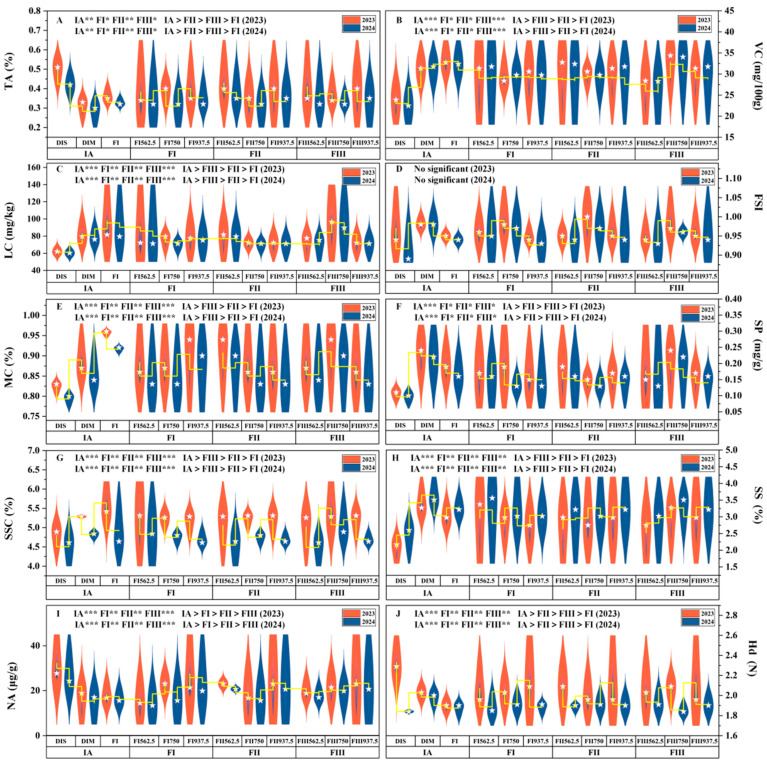
Effects of Different Factor Levels on TA (**A**), VC (**B**), LC (**C**), FSI (**D**), MC (**E**), SP (**F**), SSC (**G**), SS (**H**), NA (**I**), and Hd (**J**).

**Figure 7:**
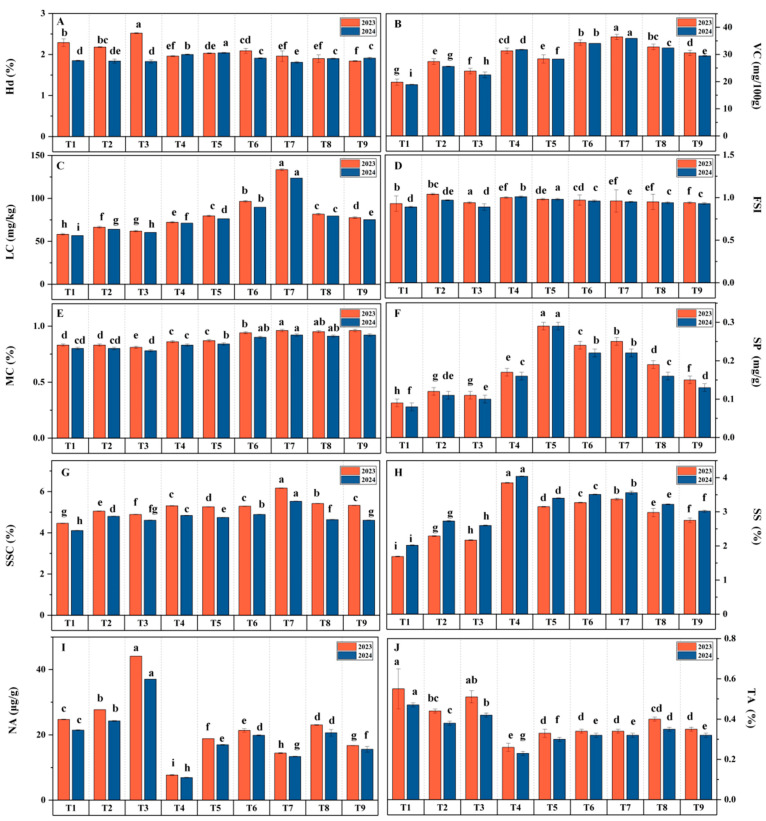
Effects of Different Treatments on Hd (**A**), VC (**B**), LC (**C**), FSI (**D**), MC (**E**), SP (**F**), SSC (**G**), SS (**H**), NA (**I**), and TA (**J**). Different letters (a–i) indicate statistically significant differences between groups.

Range analysis (Figure 6 and Figure 7) revealed that, with the exception of the fruit shape index, irrigation amount generally had a more pronounced influence on fruit quality indicators compared to fertilization amount. Notably, fertilization during the fruiting stage had a stronger impact on VC, LC, SP, and SSC than fertilization during other growth stages. The optimal water–fertilizer combinations for different quality indicators were determined as follows: VC: FI-FI 937.5, FII 750, FIII 750, LC: DIM-FI 750, FII 937.5, FIII 750, SP: FI-FI 562.5, FII 937.5, FIII 750, SSC: DIM-FI 562.5, FII 750, FIII 750, SS: FI-FI 562.5, FII 937.5, FIII 750, TA: DIS-FI 937.5, FII 562.5, FIII 562.5, MC: FI-FI 937.5, FII 562.5, FIII 750.

Compared to FI, DIS resulted in significant reductions in SSC (11.93%), SS (28.66%), LC (35.62%), SP (44.45%), and VC (30.23%). Concurrently, DIS increased TA, Hd, and NA content by 35.29%, 10.63%, and 72.67%, respectively. On the other hand, DIM led to slight decreases in SSC (4.35%), LC (7.98%), and VC (4.88%) compared to FI, but significantly enhanced SS and SP content by 12.42% and 22.22%, respectively. Overall, while DIS adversely affected multiple quality indicators of tomatoes, DIM maintained a more balanced quality profile by slightly reducing some indicators while significantly enhancing others.

### 3.3. Comprehensive Evaluation of Tomatoes

#### 3.3.1. Construction of the Comprehensive Evaluation Model for Tomatoes

**Figure 8:**
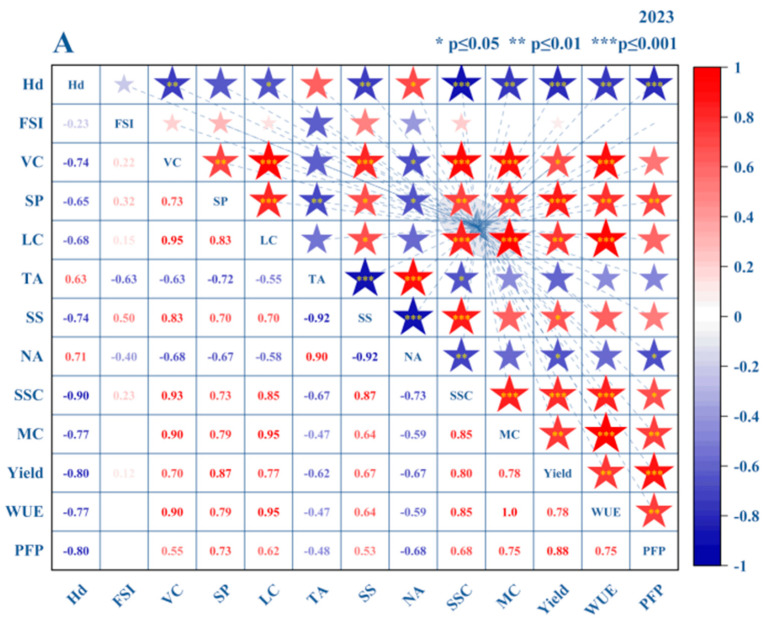

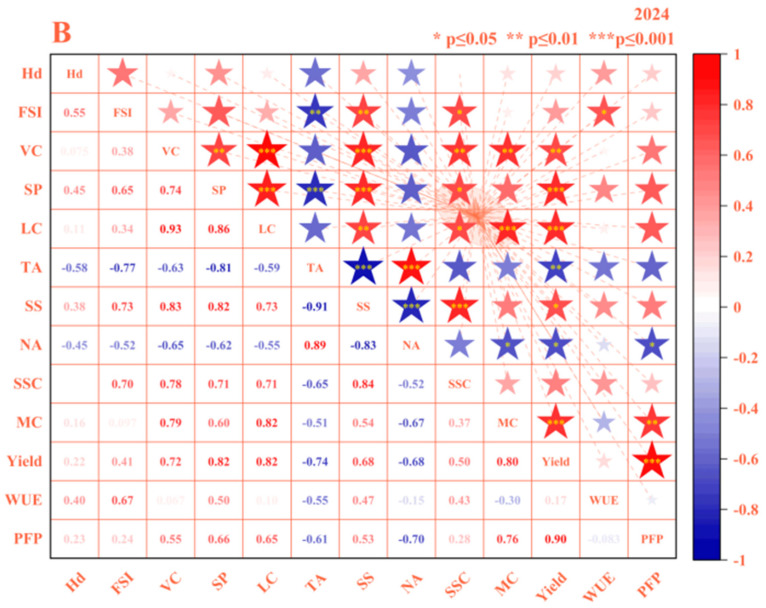
Correlation Coefficient Graphs of Quality Indicators (Hd, FSI, VC, SP, LC, TA), Yield Indicators, WUE, and PFP for 2023 (**A**) and 2024 (**B**).

During the two-year tomato growth cycle, Spearman rank correlation analysis was conducted on fruit yield, WUE, and quality-related indicators to identify the primary factors influencing fruit formation and to establish a comprehensive evaluation system for tomatoes. The study results revealed that VC exhibited significant positive correlations with LC, SS, SSC, MC, and WUE. Additionally, SP showed significant correlations with LC and Yield, and it was moderately associated with SSC, MC, and WUE. In 2023, a total of 16 significant correlations were detected, whereas in 2024, 11 significant correlations were identified (Figure 8). Although there was some overlap in the information provided by certain indicators, each metric holds substantial importance in comprehensively reflecting tomato quality and yield.

This study provides refined regulatory strategies for improving tomato quality and increasing yield through an in-depth exploration of the interrelationships among key quality and yield indicators. The findings offer valuable theoretical support and practical guidance for efficient facility cultivation management in arid and semi-arid regions. By uncovering the synergistic effects of various water–fertilizer management practices on key parameters, the study enables farmers and agronomists to optimize cultivation techniques for sustainable and high-quality tomato production. Moreover, the comprehensive evaluation model constructed in this study integrates multiple quality and yield indicators, allowing for a holistic assessment of tomato performance under different water–fertilizer coupling treatments. This model not only deepens our understanding of the complex interactions between irrigation, fertilization, and tomato physiology but also provides a robust framework for future research and agricultural practices. Ultimately, it aims to maximize both the quality and productivity of tomato crops while promoting sustainable resource use.

**Figure 9:**
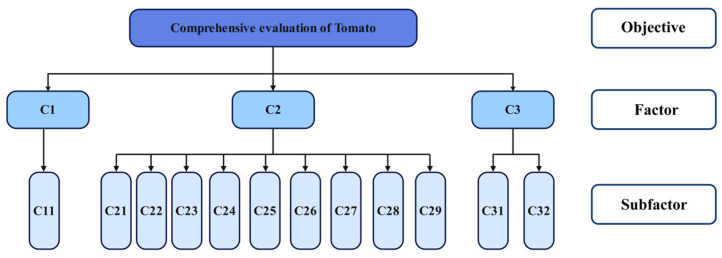
Comprehensive Evaluation Model for Tomatoes.

Although Hd exhibited negative correlations with several indicators, it remains a critical metric for assessing market acceptance, storage performance, and processing adaptability. Consequently, it was included in the evaluation indicator system. In contrast, the fruit shape index demonstrated low stability due to its sensitivity to varietal characteristics, environmental conditions, and cultivation practices, and was excluded as a key evaluation factor. Based on these considerations, a comprehensive evaluation system for tomatoes cultivated under yellow sand substrates was developed, encompassing three core dimensions: yield, quality, and efficiency (see Figure 9). Yield was represented by per hectare yield (C11). Quality was assessed using multiple indicators, including Hd (C21), VC (C22), SP (C23), LC (C24), TA (C25), SS (C26), NA (C27), SSC (C28), and MC (C29). For water–fertilizer use efficiency, WUE (C31) and PFP (C32) were selected. The hierarchical evaluation model, constructed using these indicators, provides a multidimensional assessment of tomato performance in terms of yield, quality, and resource utilization efficiency. This model offers a robust scientific framework for optimizing water–fertilizer management and supports the industrial-scale promotion of yellow sand substrate-based tomato cultivation. By systematically analyzing the interrelationships among these indicators, this study proposes refined regulatory strategies to enhance tomato quality and yield. These strategies serve as practical guidance for improving resource efficiency and achieving sustainable facility cultivation in arid and semi-arid regions.

#### 3.3.2. Analysis of the Comprehensive Scoring Method Based on the Improved Entropy Weight Method

To comprehensively and rigorously evaluate the overall performance of different treatments, this study employed a comprehensive scoring approach combined with an improved entropy weight method to determine indicator weights. Considering the inherent differences in characteristics and evaluation dimensions among quality indicators, yield, and water–fertilizer use efficiency, these categories were assessed independently. The improved entropy weight method was applied to each category to ensure objectivity and scientific accuracy in weight allocation. By performing non-negative translation on the original data, this method effectively eliminated the influence of zero values during information entropy calculations, thereby enhancing the stability and precision of weight distribution. Finally, comprehensive scores were calculated by multiplying each indicator’s score by its corresponding weight and summing the weighted values, providing an integrated evaluation of the treatments.

Using the comprehensive scoring method, the overall benefits of fruit yield, WUE, and PFP were evaluated (Table S1). The results revealed that the T5 combination achieved the highest comprehensive score across these three indicators, highlighting its superior overall performance in yield, WUE, and PFP. In the comprehensive analysis of tomato fruit quality (Table S2), the T7 combination attained the highest score, demonstrating its outstanding benefits in enhancing tomato fruit quality. These findings underscore the effectiveness of targeted water–fertilizer management strategies in achieving optimal outcomes for specific performance dimensions.

#### 3.3.3. Multi-Objective Decision Making and Evaluation Using an Improved TOPSIS Method with a Virtual Ideal Solution

This study evaluated twelve indicators, including fruit yield, WUE, PFP, VC, SP, LC, SS, SSC, Hd, TA, MC, and NA. Using a multi-criteria decision-making approach based on the TOPSIS method, the optimal water–fertilizer coupling strategy was systematically identified (Table S3).

The TOPSIS analysis results indicated that under yellow sand substrate cultivation conditions, the treatment combination T5 achieved the highest comprehensive scores in fruit yield, WUE, and PFP. This combination was identified as the best fertilization strategy for achieving high-quality and high-yield tomatoes with WUE and PFP. Specifically, this strategy involves applying 75% of the crop ET_c_ for irrigation: applying 750 kg/ha of fertilizer during the seedling stage, 937.5 kg/ha during the flowering stage, and 562.5 kg/ha during the fruiting stage.

## 4. Discussion

### 4.1. Effects of Irrigation and Fertilization on Tomato Yield, Water Use Efficiency (WUE), and Fertilizer Use Efficiency Under Yellow Sand Substrate Cultivation

Irrigation and fertilization techniques are vital for sustainable agriculture in arid regions, enabling water and fertilizer conservation while enhancing nutrient synergy and improving fertilizer use efficiency [32,33]. This study found that under the yellow sand substrate system, IA during different growth stages had a greater impact on yield, WUE, PFP, and fruit quality than fertilization factors, with IA identified as the primary determinant over the two-year experiment. Optimal IA improves soil nutrient diffusion, water absorption efficiency, and solute delivery to roots, promoting nutrient uptake [34]. However, DIS significantly reduced yield compared to FI, consistent with findings that water deficits impair cell structure, reduce photosynthesis, and limit leaf expansion, causing assimilates to prioritize root development over vegetative and reproductive growth [35,36]. Severe water stress also disrupts hormonal regulation and enzymatic activity, further inhibiting fruit yield [37]. Soil texture variations exacerbate these effects by affecting FC and PWP, restricting root-zone water availability [38]. Conversely, DIM slightly reduced yield and PFP but significantly improved WUE. Mild water stress induces osmotic adjustments, maintaining cellular structure and supporting growth under limited water conditions [39]. It also activates genes encoding osmolyte synthesis and antioxidant enzymes, enhancing cell resilience and physiological function [40]. These results highlight DIM as a promising strategy for balancing yield and resource efficiency, supporting sustainable tomato cultivation in arid environments.

Reasonable fertilization significantly improves crop yield and WUE [41]. Under DIS conditions, fruit yield showed a positive correlation with fertilization, but the benefits plateaued when fertilization exceeded a certain threshold [42]. Moderate fertilization mitigated the adverse effects of drought stress, enhancing root development and water absorption efficiency [43]. However, excessive fertilization increased soil salinity, causing osmotic stress and limiting water uptake [44]. Under FI and DIM conditions, fertilization proportions during different growth stages significantly affected fruit yield. Balanced fertilization improved water–fertilizer synergy, chlorophyll content, and photosynthetic rate, boosting energy accumulation and growth [45]. Fertilization also enhanced cell division and expansion, strengthening cell wall stability and improving fruit Hd and storability [46]. The results also indicate that T5 and T7 exhibited higher PFP, while T3 had the lowest PFP. This finding underscores the critical role of water and fertilizer balance in improving crop nutrient absorption and utilization efficiency, suggesting that the synergistic effect of water and fertilizer depends on the proper alignment of irrigation levels and fertilizer application. Adequate water supply enhances the effective transport and utilization of nutrients in the root zone, thus, improving the crop’s nutrient absorption efficiency and ultimately leading to a synergistic increase in both yield and resource use efficiency. Furthermore, fertilization during FII had the most significant impact on fruit yield and WUE, supporting floral organ development, nutrient allocation, and reproductive growth [47]. Under higher irrigation conditions, T7 achieved the highest fruit yield and PFP, while T5 exhibited the best WUE. T7, through adequate irrigation, facilitated salt leaching to a certain extent, reducing salt accumulation in the root zone and, thus, mitigating the negative impact of salt stress on crop growth. Although the changes in substrate salinity were not directly measured, the T7 treatment demonstrated an increase in yield without an abnormal rise in nitrate content, indicating that sufficient water supply not only optimized the water–fertilizer ratio but also effectively controlled salt accumulation. Therefore, it can be inferred that higher irrigation levels may have improved the salt environment in the root zone, potentially promoting crop growth and yield, thereby enhancing the synergistic effect of water and fertilizer. High fertilization during FII significantly enhanced fruit yield and WUE under FI, while DIM notably improved WUE. Stage-specific fertilization balanced vegetative and reproductive growth, optimized dry matter distribution, regulated hormone balance, and improved environmental adaptability, resulting in multi-level synergistic benefits [48]. Under yellow sand substrate cultivation, stage-specific fertilization tailored to tomato nutrient demands can improve fertilizer use efficiency, meet nutrient needs, and reduce nutrient loss [49].

This study further indicates that the T7, with sufficient irrigation and moderate fertilization, as well as the T5 with mild water deficit, exhibited higher PFP and WUE, while the T3, with insufficient water and excessive fertilization, showed the lowest PFP. These results emphasize the critical role of water and fertilizer balance in improving crop nutrient absorption and utilization efficiency, suggesting that the synergistic effect of water and fertilizer depends on the proper matching of irrigation levels and fertilizer application.

### 4.2. Effects of Irrigation and Fertilization on Tomato Fruit Quality Under Yellow Sand Substrate Cultivation

Timely nutrient supplementation is essential for enhancing fruit quality. Fertilizers in the soil must first be decomposed and transformed by soil microorganisms before being absorbed by plant roots. Excessive one-time fertilization can overstimulate microbial activity during early growth stages, disrupting nutrient decomposition and transformation in later stages and reducing nutrient availability when demand peaks [50]. Conversely, multiple topdressing applications enhance microbial metabolism, ensuring a stable and continuous nutrient supply throughout crop development [51]. This study demonstrated that fertilization during FIII significantly improved key fruit quality indicators, including TA, VC, LC, and SS. During this stage, nutrients are predominantly allocated to fruit development and maturation, promoting cell division and expansion, enhancing the activity of metabolic enzymes, and improving fruit quality [52]. The targeted nutrient supply at FIII supports the synthesis of secondary metabolites and osmotic regulatory substances, contributing to improved flavor, nutritional value, and overall fruit quality.

Compared to FI, DIS significantly reduced the content of VC, LC, MC, SP, and SS. Severe water deficits induce stomatal closure to minimize transpiration [53], limiting CO_2_ absorption and lowering photosynthetic efficiency, which ultimately reduces the production of photosynthates [54]. Water deficiency also disrupts amino acid synthesis and protein accumulation, compelling plants to prioritize essential physiological processes while downregulating non-essential protein synthesis to conserve energy and resources [55]. In contrast, DIM had minimal effects on TA, VC, LC, and SSC but increased SS, suggesting that mild water stress has limited adverse effects on fruit quality. Moreover, mild stress may promote sugar synthesis and accumulation by regulating metabolic pathways [56]. Under FI or DIM conditions, fertilization strategies during different growth stages significantly influenced TA, VC, LC, SP, SSC, and SS, highlighting the synergistic effects of reasonable fertilization and irrigation on improving fruit quality. The results indicated that T7 achieved the highest VC, LC, SSC, and SP levels, while T4 reached the highest SS content. Increasing fertilization during FIII under DIM significantly improved SS content, whereas maintaining fertilization during FI enhanced VC, LC, SSC, and SP. These findings suggest that stage-specific fertilization tailored to crop nutrient demands and irrigation conditions can optimize fruit quality and resource use efficiency under non-severe deficit irrigation.

With the advancement of facility agriculture technology, tomato cultivation in desert areas has seen a significant increase in yield. However, the market price of tomatoes remains relatively low, prompting farmers to focus not only on yield but also on improving nutritional quality and appearance. Optimizing water and fertilizer inputs has become key to improving production efficiency and resource utilization. This experiment employed a fixed fertilizer ratio and orthogonal experimental design to ensure uniform distribution of factor levels and assess the main and interaction effects of irrigation amount and fertilization strategies. Data analysis was conducted using ANOVA, range analysis, improved entropy weight method, and TOPSIS, to correct biases caused by the imbalance in fertilizer application. The results indicated that the T5 treatment performed best in terms of yield, WUE, and PFP, while the T7 treatment showed the best tomato quality. T5 was identified as the optimal fertilization strategy, effectively improving yield and quality, as well as enhancing water and fertilizer use efficiency.

However, the current orthogonal experimental design does not cover all the variables and limiting factors in different desert regions, and the fixed fertilizer ratio may not fully accommodate the dynamic nutrient needs of tomatoes at different growth stages. Due to the special characteristics of desert environments, some treatments had higher total fertilizer application across growth stages, but this design considered the low fertility and poor nutrient retention capacity of the yellow sand substrate. To further improve the study, the following approaches can be considered: (1) dynamically adjusting the fertilizer ratio based on plant physiological indicators (such as leaf nitrogen and potassium content); (2) incorporating a dynamic fertilization model, optimizing the fertilizer ratio further by using real-time monitoring data to more precisely meet the crop’s nutritional needs at different growth stages; (3) utilizing soil sensors for real-time monitoring of substrate nutrient availability to achieve precise control; (4) evaluating the long-term impact of water-saving irrigation on soil microbial communities and carbon sequestration functions. Although this study primarily focuses on water and fertilizer management for tomatoes, the findings are of certain reference value for the production management of other greenhouse crops. In arid regions, optimizing water–fertilizer coupling can improve the yield, quality, and water–fertilizer use efficiency of different crops. Therefore, in the future, this strategy can be extended to studies on other crops such as peppers and cucumbers.

## 5. Conclusions

This study reveals that, in yellow sand substrate cultivation, IA exerts a stronger impact on tomato yield, WUE, and PFP than fertilizer rates. Specifically, seedling-stage fertilization primarily influences PFP and nitrate content, flowering-stage fertilization significantly affects yield, WUE, and fruit Hd, and fruiting-stage fertilization governs the accumulation of VC, LC, and SS. By integrating an improved entropy weight method with a virtual ideal-solution-based TOPSIS model, we resolved ambiguities in traditional evaluation approaches under complex interactions, identifying DIM (75% ET_c_), coupled with staged fertilization (750 kg ha^−1^ at seedling, 937.5 kg ha^−1^ at flowering, and 562.5 kg ha^−1^ at fruiting), as the optimal strategy. Compared with conventional full irrigation, this approach reduces water and fertilizer inputs by 25% and 24%, respectively, with only a 9.4% yield loss, while boosting VC and LC contents by 18.7% and 23.6%. Although a fixed fertilizer ratio (N:P_2_O_5_:K_2_O = 20%:20%:20%) may constrain potassium supply during peak fruiting and the experiment was limited to arid conditions in a yellow sand substrate, these findings present a transferable water–fertilizer synergy framework for greenhouse cultivation in arid regions. Future research should employ real-time monitoring technologies to refine fertilizer ratios dynamically and confirm the approach’s feasibility across diverse environments, thereby supporting national water-saving and yield-increasing initiatives.

## Figures and Tables

**Table 1:** Amounts of irrigation water and fertilizer applied.

Treatment	Irrigation (m^3^ ha^−1^)		Fertilization Rate (kg ha^−1^)—FI	Fertilization Rate (kg ha^−1^)—FII	Fertilization Rate (kg ha^−1^)—FIII
	2023	2024			
T1 (DISFI562.5FII562.5FIII562.5)	1525	1475	562.5	562.5	562.5
T2 (DISFI750FII750FIII750)	1525	1475	750	750	750
T3 (DISFI937.5FII937.5FIII937.5)	1525	1475	937.5	937.5	937.5
T4 (DIMFI562.5FII750FIII937.5)	2287.5	2212.5	562.5	750	937.5
T5 (DIMFI750FII937.5FIII562.5)	2287.5	2212.5	750	937.5	562.5
T6 (DIMFI937.5FII562.5FIII750)	2287.5	2212.5	937.5	562.5	750
T7 (FIFI562.5FII937.5FIII750)	3050	2950	562.5	937.5	750
T8 (FIFI750FII562.5FIII937.5)	3050	2950	750	562.5	937.5
T9 (FIFI937.5FII750FIII562.5)	3050	2950	937.5	750	562.5

**Table 2:** Basic Physical and Chemical Properties of Yellow Sand Substrate.

Year	Ammonium Nitrogen (mg/kg)	Available Phosphorus (mg/kg)	Available Potassium (mg/kg)	Maximum Field Capacity (%)	Yellow Sand Substrate Porosity (%)	Water-Holding Porosity (%)	Soil Bulk Density (g/cm^3^)	pH
2023	108.24	93.57	236.77	18.3	37.1	17.6	1.61	7.82
2024	101.37	91.52	258.6	17.6	36.7	18.1	1.53	7.73

## Data Availability

The original contributions presented in this study are included in the article/Supplementary Materials. Further inquiries can be directed to the corresponding authors.

## Supplementary Materials

The following supporting information can be downloaded at: https://www.mdpi.com/article/10.3390/plants14060936/s1, Table S1: Comprehensive Scoring Analysis of Tomato Yield, WUE, and PFP. Table S2: Comprehensive Scoring Analysis of Tomato Quality; Table S3: Analysis of Tomato Indicators Using the Improved TOPSIS Method with Virtual Ideal Solutions.
