# Supplementary material for: Optimizing Water–Fertilizer Coupling Across Different Growth Stages of Tomato in Yellow Sand Substrate: Toward Enhanced Yield, Quality, and Resource Use Efficiency
Source: Plants (Basel). 2025 Mar 17;14(6):936. doi: 10.3390/plants14060936 (PMC11946079; doi:10.3390/plants14060936)
Supplement: Supplementary file 1 [file plants-14-00936-s001.zip › plants-3498433-supplementary.pdf]

Optimizing Water-Fertilizer Coupling Across Different Growth Stages of Tomato in Yellow-Sand Substrate: Toward Enhanced Yield, Quality, and Resource Use Efficiency

Yalong Song[1,2,4], Jiahui Xu[1,2,4], Shuo Zhang[1,2,4], Jianfei Xing[1,2,3,4*], Long Wang[1,2,4], Xufeng Wang[1,2,4], Can Hu[1,2,4], Wentao Li[1,2,4], Zhanming Tan[5], Yunxian Cheng[5]

[1] College of Mechanical and Electrical Engineering, Tarim University, Alar 843300, China;
[2] Xinjiang Production and Construction Corps Key Laboratory of Utilization and Equipment of Special
[3] College of Engineering, China Agriculture University, Beijing 100083, China
[4] Modern Agricultural Engineering Key Laboratory, Universities of Education Department of Xinjiang Uygur Autonomous Region, Alar 843300, China.
[5] College of Horticulture and Forestry Sciences, Tarim University, Alar, China.
* Correspondence: 120200012@taru.edu.cn;

The supplementary documents for this article are as follows:

Table S1. Comprehensive Scoring Analysis of Tomato Yield, WUE, and PFP.

Year	Treatment	IA	FA			Membership Degree Value			Comprehensive Score
			FI	FII	FIII	Yield	WUE	PFP	
2023	T1	DIS	562.5	562.5	562.5	0.00	0.00	0.28	0.0030
	T2	DIS	750	750	750	0.15	0.28	0.20	0.0084
	T3	DIS	937.5	937.5	937.5	0.20	0.62	0.00	0.0124
	T4	DIM	562.5	750	937.5	0.32	0.55	0.32	0.0157
	T5	DIM	750	937.5	562.5	0.66	1.00	0.94	0.0326
	T6	DIM	937.5	562.5	750	0.27	0.37	0.24	0.0115
	T7	FI	562.5	937.5	750	1.00	0.25	1.00	0.0253
	T8	FI	750	562.5	937.5	0.48	0.19	0.75	0.0160
	T9	FI	937.5	750	562.5	0.44	0.03	0.51	0.0107
2024	T1	DIS	562.5	562.5	562.5	0.00	0.00	0.33	0.0029
	T2	DIS	750	750	750	0.14	0.26	0.26	0.0085
	T3	DIS	937.5	937.5	937.5	0.05	0.37	0.00	0.0070
	T4	DIM	562.5	750	937.5	0.32	0.53	0.38	0.0163
	T5	DIM	750	937.5	562.5	0.69	1.00	0.97	0.0336
	T6	DIM	937.5	562.5	750	0.26	0.35	0.30	0.0121
	T7	FI	562.5	937.5	750	1.00	0.26	1.00	0.0266
	T8	FI	750	562.5	937.5	0.48	0.20	0.77	0.0164
	T9	FI	937.5	750	562.5	0.48	0.06	0.58	0.0127
2023	K1	0.0239	0.0440	0.0305	0.0464	The weights of each indicator are:			
	K2	0.0598	0.0571	0.0349	0.0453	Yield			0.0514
	K3	0.0520	0.0347	0.0703	0.0440	WUE			0.0494
	Range value	0.0120	0.0074	0.0133	0.0008	PFP			0.0353
	Factors priority order	FII＞IA＞FIII＞FI							
	Optimal combination	DIMFI750FII937.5FIII562.5							
2024	K1	0.0184	0.0458	0.0314	0.0492				
	K2	0.0620	0.0585	0.0585	0.0473				
	K3	0.0558	0.0319	0.0319	0.0397				
	Range value	0.0146	0.0089	0.0090	0.0032				
	Factors priority order	IA＞FII＞FI＞FIII							
	Optimal combination	DIMFI750FII937.5FIII562.5							

Table S2. Comprehensive Scoring Analysis of Tomato Yield, WUE, and PFP.

Year	Treatment	IA	FA			Membership Degree Value									Comprehensive Score
			FI	FII	FIII	VC	SP	LC	SS	SSC	Hd	TA	MC	NA	
2023	T1	DIS	562.5	562.5	562.5	0.00	0.00	0.00	0.00	0.00	0.66	1.00	0.13	0.47	0.0279
	T2	DIS	750	750	750	0.46	0.13	0.11	0.28	0.35	0.50	0.62	0.13	0.55	0.0309
	T3	DIS	937.5	937.5	937.5	0.25	0.08	0.05	0.22	0.25	1.00	0.86	0.00	1.00	0.0398
	T4	DIM	562.5	750	937.5	0.69	0.40	0.19	1.00	0.50	0.18	0.00	0.33	0.00	0.0311
	T5	DIM	750	937.5	562.5	0.51	1.00	0.28	0.68	0.47	0.28	0.26	0.40	0.31	0.0430
	T6	DIM	937.5	562.5	750	0.87	0.75	0.51	0.73	0.49	0.37	0.29	0.87	0.38	0.0541
	T7	FI	562.5	937.5	750	1.00	0.80	1.00	0.78	1.00	0.18	0.29	1.00	0.19	0.0799
	T8	FI	750	562.5	937.5	0.78	0.48	0.31	0.60	0.56	0.09	0.48	0.93	0.42	0.0440
	T9	FI	937.5	750	562.5	0.65	0.30	0.26	0.49	0.51	0.00	0.31	1.00	0.25	0.0350
2024	T1	DIS	562.5	562.5	562.5	0.00	0.00	0.00	0.00	0.00	0.16	1.00	0.13	0.48	0.0183
	T2	DIS	750	750	750	0.39	0.13	0.11	0.35	0.49	0.12	0.63	0.13	0.58	0.0245
	T3	DIS	937.5	937.5	937.5	0.21	0.08	0.05	0.29	0.35	0.09	0.80	0.00	1.00	0.0234
	T4	DIM	562.5	750	937.5	0.76	0.35	0.22	1.00	0.51	0.83	0.00	0.33	0.00	0.0457
	T5	DIM	750	937.5	562.5	0.55	1.00	0.29	0.68	0.44	1.00	0.28	0.40	0.33	0.0559
	T6	DIM	937.5	562.5	750	0.89	0.67	0.49	0.74	0.55	0.39	0.36	0.87	0.43	0.0540
	T7	FI	562.5	937.5	750	1.00	0.68	1.00	0.77	1.00	0.00	0.35	1.00	0.21	0.0668
	T8	FI	750	562.5	937.5	0.79	0.36	0.34	0.59	0.37	0.37	0.51	0.93	0.46	0.0478
	T9	FI	937.5	750	562.5	0.64	0.23	0.28	0.50	0.35	0.42	0.38	1.00	0.29	0.0415
2023	K1	0.0986	0.1389	0.1259	0.1059					The weights of each indicator are:					
	K2	0.1282	0.1179	0.1179	0.1649					VC		0.029			

	K3	0.1589	0.1289	0.1627	0.1148	SP	0.052
	Range value	0.0201	0.0070	0.0149	0.0197	LC	0.057
	Factors priority order	IA＞FIII＞FII＞FI				SS	0.027
	Optimal combination	FIFI562.5FII937.5FIII750				SSC	0.026
	K1	0.0662	0.1308	0.1201	0.1157	Hd	0.056
	K2	0.1557	0.1283	0.1117	0.1454	TA	0.030
	K3	0.1561	0.1189	0.1461	0.1169	MC	0.048
2024	Range value	0.0300	0.0040	0.0115	0.0099	NA	0.032
	Factors priority order	IA＞FII＞FIII＞FI					
	Optimal combination	FIFI562.5FII937.5FIII750					

Table S3. Analysis of Tomato Indicators Using the Improved TOPSIS Method with Virtual Ideal Solutions.

Year	Treatment	IA	FA			Distance				Weighted Comprehensive Distance		C_i	Ranking
			FI	FII	FIII	Z^+	Z^-	V^+	V^-	S^+	S^-		
2023	T1	DIS	562.5	562.5	562.5	0.2450	0.0310	0.4935	0.1457	0.2698	0.0425	0.1360	9
	T2	DIS	750	750	750	0.2140	0.0530	0.4750	0.1502	0.2401	0.0627	0.2071	7
	T3	DIS	937.5	937.5	937.5	0.2370	0.0420	0.4611	0.1728	0.2594	0.0551	0.1751	8
	T4	DIM	562.5	750	937.5	0.1270	0.1870	0.4724	0.1380	0.1615	0.1821	0.5299	2
	T5	DIM	750	937.5	562.5	0.0940	0.2000	0.4119	0.1981	0.1258	0.1998	0.6137	1
	T6	DIM	937.5	562.5	750	0.1480	0.1380	0.4421	0.1660	0.1774	0.1408	0.4425	4
	T7	FI	562.5	937.5	750	0.1260	0.1760	0.4265	0.1867	0.1561	0.1771	0.5315	3
	T8	FI	750	562.5	937.5	0.1600	0.1210	0.4489	0.1619	0.1889	0.1251	0.3984	5
	T9	FI	937.5	750	562.5	0.1630	0.1110	0.4708	0.1319	0.1938	0.1131	0.3685	6
2024	T1	DIS	562.5	562.5	562.5	0.2530	0.0350	0.4988	0.1533	0.2776	0.0468	0.1443	9
	T2	DIS	750	750	750	0.2220	0.0550	0.4775	0.1581	0.2475	0.0653	0.2088	7
	T3	DIS	937.5	937.5	937.5	0.2460	0.0390	0.4780	0.1723	0.2692	0.0523	0.1628	8
	T4	DIM	562.5	750	937.5	0.1350	0.1840	0.4614	0.1471	0.1676	0.1803	0.5182	3
	T5	DIM	750	937.5	562.5	0.1100	0.1930	0.4109	0.2108	0.1401	0.1948	0.5817	1
	T6	DIM	937.5	562.5	750	0.1560	0.1360	0.4379	0.1710	0.1842	0.1395	0.4310	4
	T7	FI	562.5	937.5	750	0.1260	0.1940	0.4109	0.2028	0.1545	0.1949	0.5578	2
	T8	FI	750	562.5	937.5	0.1730	0.1130	0.4443	0.1654	0.2001	0.1182	0.3714	5
	T9	FI	937.5	750	562.5	0.1800	0.0990	0.4612	0.1486	0.2081	0.1040	0.3331	6
